# Stress Corrosion Cracking of Friction Stir-Welded AA-2024 T3 Alloy

**DOI:** 10.3390/ma13112610

**Published:** 2020-06-08

**Authors:** Marina Cabrini, Sara Bocchi, Gianluca D’Urso, Claudio Giardini, Sergio Lorenzi, Cristian Testa, Tommaso Pastore

**Affiliations:** 1 Department of Engineering and Applied Sciences, University of Bergamo, 24044 Dalmine (BG), Italy; marina.cabrini@unibg.it (M.C.); cristian.testa@unibg.it (C.T.); tommaso.pastore@unibg.it (T.P.); 2 National Interuniversity Consortium of Materials Science and Technology (INSTM) Research Unit of Bergamo, 24044 Dalmine (BG), Italy; 3 Center for Colloid and Surface Science (CSGI) Research Unit of Bergamo, 24044 Dalmine (BG), Italy; 4 Information and Production Engineering, Department of Management, University of Bergamo, 24044 Dalmine (BG), Italy; sara.bocchi@unibg.it (S.B.); gianluca.d-urso@unibg.it (G.D.); claudio.giardini@unibg.it (C.G.)

**Keywords:** stress corrosion cracking, slow strain rate test, constant load test, friction stir welding (FSW), AA2024

## Abstract

The paper is devoted to the study of stress corrosion cracking phenomena in friction stir welding AA-2024 T3 joints. Constant load (CL) cell and slow strain rate (SSR) tests were carried out in aerated NaCl 35 g/L solution. During the tests, open circuit potential (OCP) and electrochemical impedance spectroscopy (EIS) were measured in the different zones of the welding. The results evidenced initial practical nobilty of the nugget lower compared to both heat-affected zone and the base metal. This effect can be mainly ascribed to the aluminum matrix depletion in copper, which precipitates in form of copper-rich second phases. In this zones, no stress corrosion cracking was noticed, but well-evident stress-enhanced intergranular corrosion occurred. This is due to the uneven distribution of platic deformation during the slow strain rate tests. Higher strain values are localized at the heat affected zone, where softening occurs. On the contrary, stress values at the nugget are not sufficient to favor both the initiation and propagation of stress corrosion cracks. In the range of processing parameter studied in this experimental work, the stress corrosion cracking susceptibility of the friction stir welding (FSW)-ed alloy is then similar to that of the base metal.

## 1. Introduction

Friction stir welding (FSW) is a solid-state joining process that is gaining a lot of attention especially for high-strength age-hardening aluminum alloys, in which mechanical properties are strictly dependent upon heat treatments at relatively lower temperatures [1,2,3,4]. The joint is produced by the friction generated between a rotating pin put directly in contact with the base metal. The temperature of the material increases significantly in the contact zone without melting the material itself [5]. The increase in temperature increases the material deformability, thus allowing the mixing of the two sides of the joint [6]. The thermo-mechanical action causes the material recrystallization that is localized in the central area of the weld, called nugget characterized by outstanding tensile properties [7,8]. The zones close to the nugget, which has the microstructure altered both by the action of the mechanical straining and the thermal effect, is called thermo-mechanically affected zone (TMAZ); after this zone, moving away from the nugget toward the base metal, there is an area that has not undergone mechanical straining, but only heating and therefore it is called heat-affected zone (HAZ). The microstructure and extension of these zone, nugget, TMAZ, and HAZ, depend on the size and shape of the pin, the thickness of the sheet, and the welding parameters, in particular the force exerted on the pin, its rotation speed, and feed rate [9,10]. Depending on the tool rotation side, “advancing side” and “retreating side” are defined [11]. Lot of efforts have been paid to study the effect of the tool rotation on both the extension of these zones and the effects on mechanical properties in function of the process parameters, i.e., speed and feed rate [12,13,14]. Among all the aluminum alloys, series 2XXX, 6XXX [15], and 7XXX [16,17] are the most affected by the microstructure and mechanical properties modification induced by FSW, because their resulting microstructure is a combination between the original temper and the thermomechanical alterations induced by FSW process. Microstructural modifications are relevant across the welding and both mechanical and corrosion behavior [18] at the nugget, TMAZ, and the HAZ are strictly dependent upon the process parameters.

The combined action of applied stress and corrosive environment can also promote stress corrosion cracking (SCC) in susceptible alloys [19,20,21,22] and the effect of the microstructural modification induced by FSW on the intrinsic susceptibility of such alloys is yet far to be completely understood. The higher number of works are on the SCC of alloy 7XXX but, in the knowledge of the authors, only few papers reported the SCC behavior of FSW joints in AA-2024. 

Wang et al. reported SCC insurgence on FSW AA-2024-T3 in the interface between the nugget and the TMAZ in 3.5 wt.% NaCl solution. The stress corrosion cracks were nucleated by pitting. The SCC susceptibility of the FSWed joints increases with increasing travel speed, owing to the increase of the size of second phase particles. A SCC mechanism controlled by the metal anodic dissolution at the OCP and hydrogen accelerated metal embrittlement in the active zones was suggested [23] In a previous paper of the authors, no stress corrosion cracking was observed on four point bent beam specimens after 1500 h of exposure in NaCl 35 g/L, but the intergranular corrosion attack became penetrating in the presence of loading, giving rise to stress-enhanced intergranular corrosion of the alloy [14]. 

The aim of this work is to study the stress corrosion cracking susceptibility of FSWed AA-2024 T3 aluminum alloy. Electrochemical tests and free corrosion potential measurements were carried out during SCC test to assess corrosion phenomena occurring during exposure.

## 2. Materials and Methods

### 2.1. Preparation of the Welds

**Figure 1:**
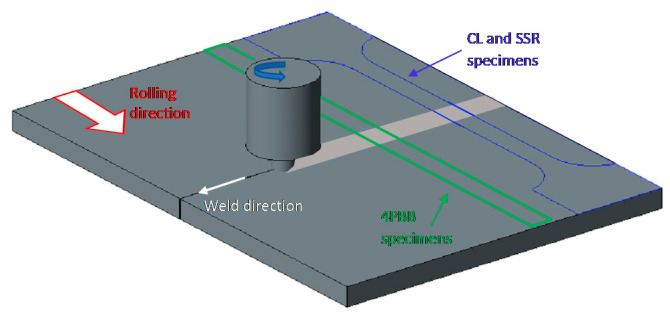
Schematic representation of the friction stir welding (FSW) and the machining of the specimens.

Aluminum sheets (size 200 mm × 80 mm × 4 mm thickness) were welded by using a tool with smooth plane shoulder (16 mm diameter) and pin having a frustum of cone shape (pin maximum and minimum diameters equal to 6 and 4 mm, height equal to 3.8 mm) (Figure 1). The rotational speed (S) of the tool was fixed at 1500 rpm and feed rate (F) was 10 mm/min. The set-up of the welding process was reported in previous works by authors [14,18,22,24]. The mechanical properties of the base materials and the welded joints are shown in Table 1. For the FWSed specimens, only the ultimate tensile strength (UTS) is reported, because it was not possible to evaluate the yield strength (YS) owing to the localization of the plastic straining of the specimens in the TMAZ/HAZ zone. The decrease in the mechanical properties at the thermomechanical and heat affected zones is the result of the modification in the microstructure of the alloy, with solubilization and reprecipitation of the straightening second phases, as reported in previous works by authors [10,14,18,22,24]. A good reproducibility was observed in the tensile strength of the FSWed joints, thus confirming the absence of macroscopic defects alongside the weld.

### 2.2. Constant Load Tests

**Figure 2:**
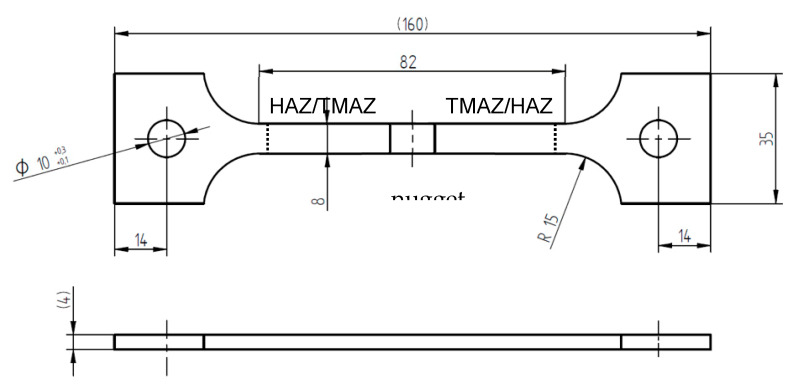
FWSed specimen used in constant load (CL) e SSR test; the specimens of the slow strain rate (SSR) test on base materials have the same dimension without weld.

**Figure 3:**
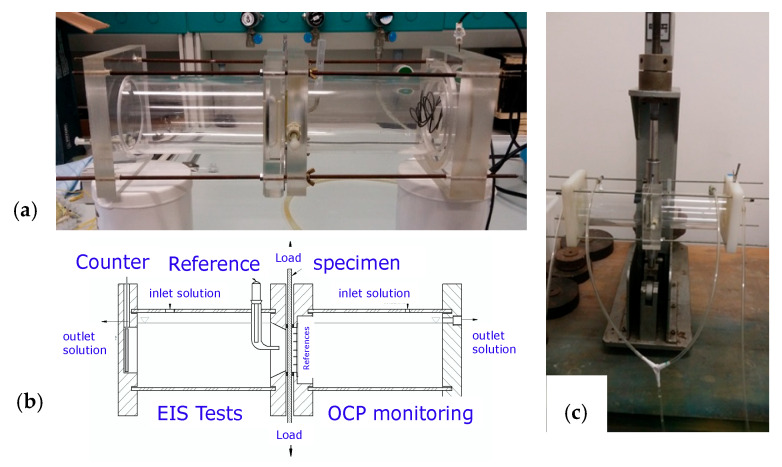
(**a**) Image of the cell used for the CL tests. (**b**) Schematic illustration of the test apparatus and (**c**) image of one specimen under the CL testing machine.

Constant load (CL) tests were carried out on dog bone shaped specimens with 8 mm width, 4 mm thickness, and 82 mm gage length with the weld positioned at the center of the gage length (Figure 2). The specimens were inserted in a double compartment cell with a window to expose about 50% of the central area (Figure 3a,b). All the compartments were filled with aerated 35 g/L NaCl solution, at 25 °C. The solution was constantly recirculated by means of a pump. The configuration of the cell was chosen in order to monitor one side the open circuit potential (OCP) and carry out the electrochemical impedance spectroscopy (EIS) test on the other side at the same time.

The OCP monitoring was done by means of homemade three Ag/AgCl references electrodes with E = +0.200 V vs. NHE (normal hydrogen electrode). The results were presented after conversion as the saturate calomel electrode (SCE-E = +0.240 V vs. NHE, Amel Instrument, Milano, Italy). The data were registered by a Ivium CompactStat instrument; the sampling frequency was varied during the experiment from one measurement per second in the initial instants and during the loading phases of the specimen to a 10-h monitoring, with acquisition of one point per minute every 100 or 200 h, during maintenance of the constant load of the specimens. The EIS tests were performed using an Ivium CompactStat instrument. Sinusoidal signal with 0.010 V amplitude in the frequencies range between 10 kHz and 10^−2^ Hz was applied. EIS tests were carried out on the CL speciemens just dipped, after 24, 48 (before and after loading the specimen), 480, 504 (before anf after increase the load on the specimen), 552, and 650 h (before and after to un-load the specimen). 

The specimens were loaded by means of a tensile testing machine with a double lever arm (Figure 3c), according to NACE TM 0177-2005 Method A, the loads were imposed by adding weights to the lever, the lever force had previously been calibrated through a calibrated load cell with 10 N accuracy. The testing procedure is as following. The specimen was initially positioned in the cell, the testing solution was inserted, and the first EIS spectrum was registered. The second EIS spectrum was measured after 24 h, the third after 48 h; after the third EIS measurement, the specimen was loaded at 50–55% UTS of the FSWed joints, and the EIS measure immediately repeated. This load was chosen because it is practically in the correspondence of the deviation from the linearity of the stress–strain curve of the weld; it could be assumed that, under this stress, the less resistance zone of the gauge length becomes plastically deformed, while the other parts of the specimen remain in elastic straining. The monitoring of OCP and EIS spectrum continued for 504 h, after these the load was further increased in order to achieve a stress value of about the 80% of the ultimate tensile strength of the weld, thus achieving uniform plastic deformation conditions along the gage length of the specimen. The load was then maintained for 650 h. 

At the end of the exposure period, the specimens were unloaded, cleaned, and observed under the scanning electron microscope (SEM, Zeiss EVO 40, Oberkochen, Germany) with energy dispersive spectroscopy (EDS) Oxford X-Act in order to analyze the corrosion products morphology and composition. Metallographic cross sections along the longitudinal direction were also analyzed. The gauge length was cut from the specimen, then longitudinally sectioned using a metallographic cutting machine (Metkon Servocut, Bursa, Turkey) in the central part. The section was embedded in resin and grinded with emery paper and polished with colloid alumina until 0.1 μm of roughness. The metallographic section was observed by a Nikon optical microscope and the SEM without metallographic attack and after attack with Keller reagent. 

### 2.3. Slow Strain Rate Tests

The slow strain rate (SSR) tests were carried out by using specimens of the same dimensions as for CL tests. The SSR tests were also performed on AA-2024 T3 base material for comparison.

The tests were carried out by means of a homemade SSR testing machine with four independent loading stations. The load is applied through an electric motor and reduction gears that can impose displacement rates between 5 × 10^−7^ and 5 × 10^−3^ mm/s. The calibration of the cells is performed independently. Acquisition system (spider 8 HBM Italy, Milano, Italy) read the load and a function of time; the displacement of the tensile grip is evaluated multiplying the displacement rate for the time. A displacement rate of 8.2 × 10^−4^ mm/s, corresponding to 10^−6^ s^−1^ average strain rate along the gage length, was chosen. SSR tests were carried out according to the ASTM 129-00 (2013), exception for the specimen’s geometry that was changed to test all the weld. The cell was like those used for the CL tests, with the same configuration of the electrodes, but smaller than that for CL tests, to be settled under the SSR machine, filled with an aerated solution of 35 g/L NaCl. No EIS spectra were obtained during this test because the OCP did not remain constant. At the end of the tests, the specimens were washed in distilled water and rinsed in acetone to permit the observation of the fracture surface under the SEM. Then the specimens were longitudinally sectioned for the metallographic observation with the same procedure illustrated for the CL test. 

### 2.4. Electrochemical Characterizations of the Different Zones of the Weld

**Figure 4:**
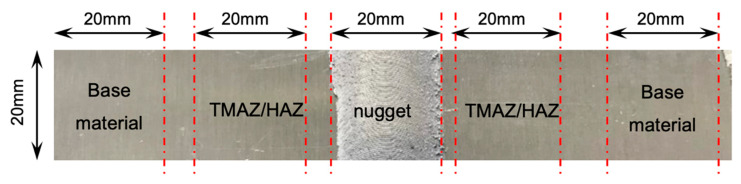
Scheme of the sampling of the different areas of the welding and of the base metal for the monitoring of open circuit potential (OCP) and electrochemical impedance spectroscopy (EIS).

For comparison, EIS and OCP measurements were performed on the separated different zones of the FSW joints: the nugget, the HAZ/TMAZ (it was not possible to cut the specimens separating these zones), and the base material in unloaded condition. The weld was cut as illustrated in Figure 4; the isolate specimens with the nugget, the TMAZ/HAZ zone, and the base material were connected with an external wire and then cold mounted with epoxy resin. The exposed surface of the specimen was 20 × 20 mm^2^. It was grounded with emery paper and then polidhed with colloidal alumina up to 0.3 μm. The specimens were dipped in separate glass cells in aerated 35 g/L NaCl (Carlo Erba, RPA grade, Cornaredo, Italy) solution. Standard saturated calomel electrode (SCE E = +0.240 V vs. NHE, Amel Instrument, Milano, Italy) was positioned at the surface of the specimens by using a Luggin capillary to compensate the ohmic drop in the electrolyte. Electrochemical impedance spectroscopy (EIS) tests were performed with the same procedure used for on the CL test. 

## 3. Results and Discussion

**Figure 5:**
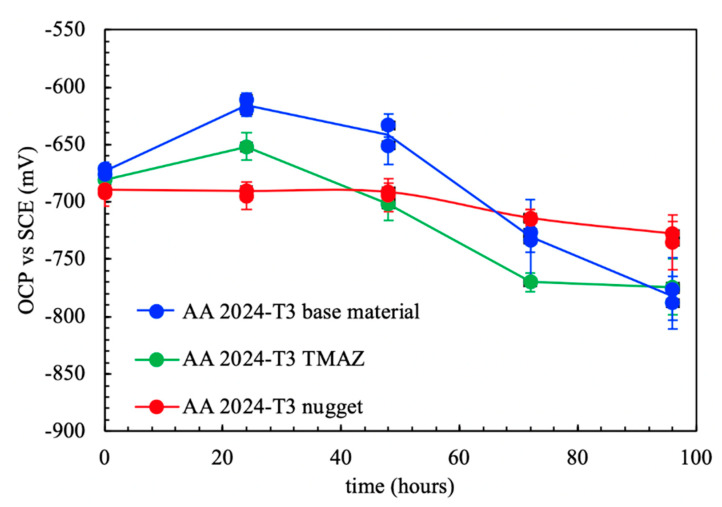
OCP in function of exposure time of the different zones of AA-2024 T3 FSWed joint in aerated NaCl 0.6 M at 23 °C.

Figure 5 shows the OCP measurements in aerated NaCl solution at the nugget, the HAZ/TMAZ, and the base material with regard to exposure time.

Similar values of the OCP were measured on the three different zones just after dipping. The nugget showed constant OCP values during all the exposure periods. Different behavior was observed for the base material and the heat-affected zones (HAZ/TMAZ), which showed an initial increase of OPC followed by a decrease of about 150 mV. 

**Figure 6:**
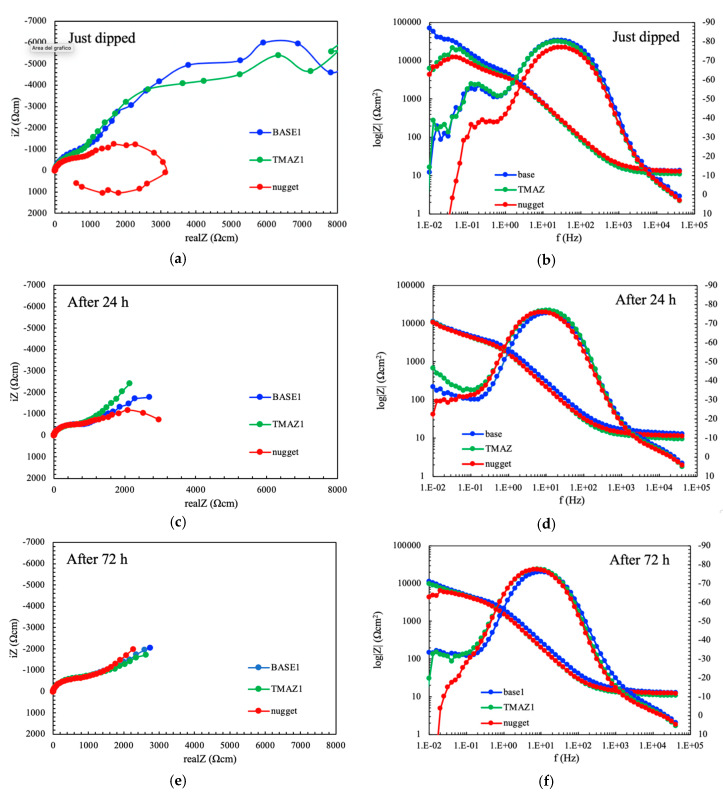
EIS spectra (**a**), (**c**), and (**e**) Nyquist and (**b**), (**d**), and (**e**) Bode representation, of the separate different zones of the weld; (**a**) and (**b**) just dipped; (**c**) and (**d**) after 24 h; (**e**) and (**f**) after 72 h of immersion in aerated NaCl 0.6 M solution.

Figure 2 shows the EIS spectra for the specimens obtained from the different zones of the weld with regard to exposure time. The spectra collected at the nugget are quite different compared to the base material and TMAZ just after dipping. The Nyquist plot shows two overlapped capacitive loops, and an inductive one at very low frequencies, whereas the base material and the TMAZ presented a capacitive loop and diffusive behavior at very low frequencies (Figure 6a). The Bode diagram (Figure 6b) shows the highest value of the impedance modulus of the base materials, indicating a stable passive condition. Conversely, the values of the impedance modulus of the nugget and the TMAZ decrease at the low frequency, thus indicating activation of the metal surface during low frequency measurements. All the specimens showed two phase constants, one at intermediate frequency and the other at very low frequencies.

After only 24 h of immersion, the shape of EIS spectra significantly changed (Figure 6c,d). The amplitude of the EIS loops in Nyquist plot decreased significantly for all the different zones of the weld. Diffusive behavior was still observed for the base metal and the TMAZ, while a second capacitive loop can be distinguished for the nugget—but flattened—and the inductive loop disappeared. All the specimens showed only one phase constant, but the frequency range is lower compared to just dipped conditions. An increase in phase can be noticed at very low frequency for the base material and the TMAZ, thus indicating that a second phase constant could be present at very low frequencies. The impedance modulus achieved the values of 10^4^ Ωcm^2^ for all the zones of the weld, meaning that activation occurred also on base metal and the TMAZ.

After 72 h of immersion, the EIS spectra became practically overlapped, with only a slight difference between the impedance modulus of the nugget with respect to the base material and the TMAZ (Figure 6e,f). Well evident diffusive behavior can be noticed at very low frequencies because of the presence of corrosion products deposit.

These results seem to evidence an initial higher activity of the nugget with respect to the other zones, but these differences become negligible at longer exposures. After the tests, all the specimens showed general corrosion morphologies. 

**Figure 7:**
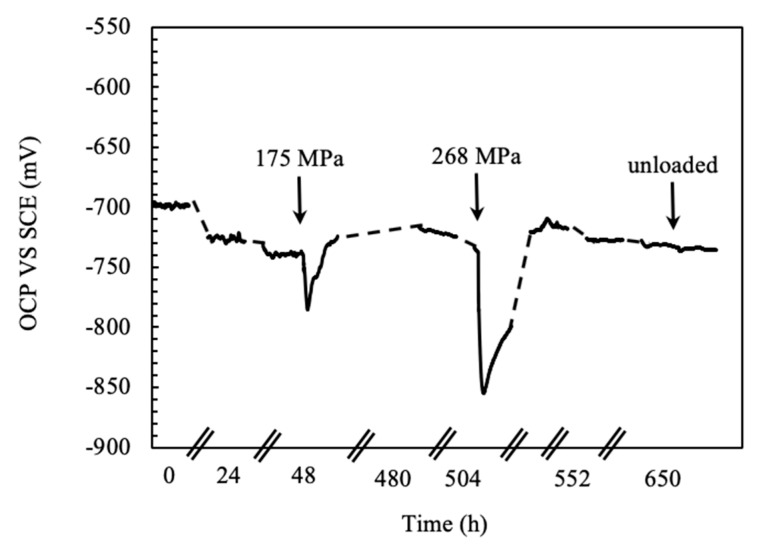
OCP measurements during 720 h exposure of CL specimen of the FSWed AA-2024 T3 alloy in aerated NaCl 0.6 M at 23 °C.

The behavior significantly modifies as loading is applied. The corrosion potential showed sudden decrease at the application of monoaxial constant load, as reported in Figure 7. The effect is attributable to the breakdown of the thick, porous, and not adherent scale of aluminum oxide formed on specimens after prolonged exposures, which exposes the aluminum matrix directly to the aggressive environment. The potential approached the steady state value—i.e., the value in unloading conditions—because of self-healing effect of corrosion products of aluminum. The decrease in the corrosion potential is more pronounced when the specimens are strained in the plastic field. 

**Figure 8:**
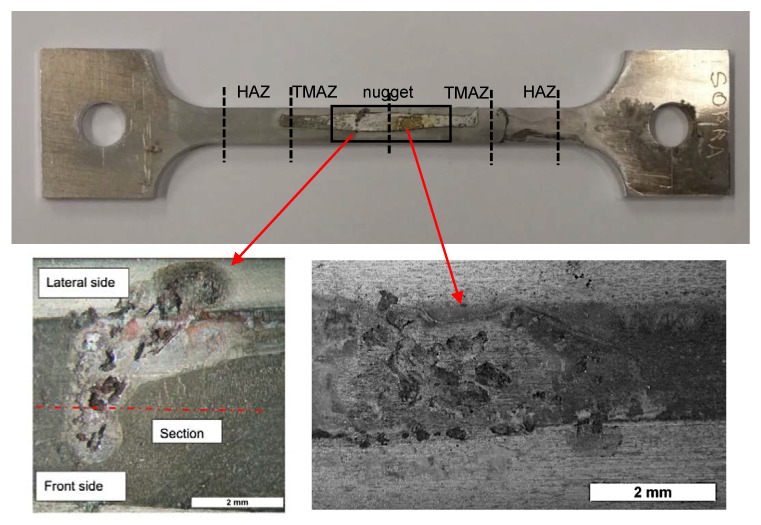
Corrosion morphology of FSWed AA-2024 T3 alloy after CL test in aerated 35 g/L NaCl.

**Figure 9:**
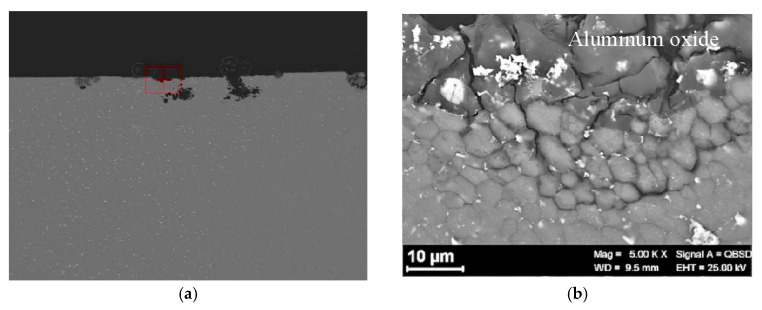
Metallographic section of Figure 6: (**a**) Macro image of the nugget with several localized attacks; (**b**) close-up of the red zone in (**a**).

Specimens failure was not observed under constant loading for 650-h exposure. The analysis of the specimens after the tests showed an intergranular attack in the nugget near the zone of beginning of the TMAZ (Figure 8 and Figure 9).

**Figure 10:**
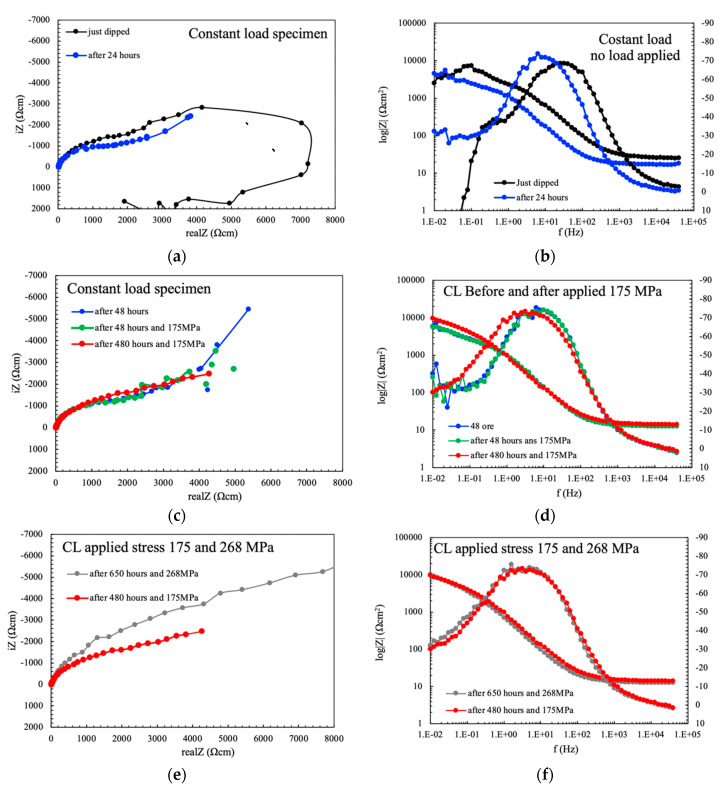
EIS spectra (**a**), (**c**), and (**e**) Nyquist representation, (**b**), (**d**), and (**e**) Bode representation of the constant load specimen without load just dipped and after 24 h (**a**,**b**), after 48 h without load, and after 175 MPa, and after 480 h loaded at 175 MPa (**a**,**d**) and after 480 h at 175 MPa and 650 h at 268 MPa (**e**,**f**) in aerated NaCl 0.6 M solution.

The EIS spectra collected on CL specimens showed certain different behavior just after dipping and after 24 h (Figure 10a,b). The spectra mainly approach that of the nugget (compare Figure 10a,b with Figure 6a,b). It can then be stated that EIS tests carried out during CL tests are mainly affected by the most active zone of the joint—i.e., the nugget. The specimen just after dipping presents two overlapped capacitive loops and an inductive one (Figure 10a)—typical of non-stationary OCP values during early exposures EIS tests (Figure 10b). After 24 h, the Nyquist diagram presented a well-evident diffusive behavior (compare Figure 10c,d to Figure 6c,d). The increase in the load does not modify the EIS spectra, that are overlapped to the condition without applied load after 24-h exposure (Figure 10c–f), thus confirming that the corrosion process at longer exposures is only limited by the presence of the pseudo-passive film by corrosion products that are able to heal the cracks promoted by loading.

**Figure 11:**
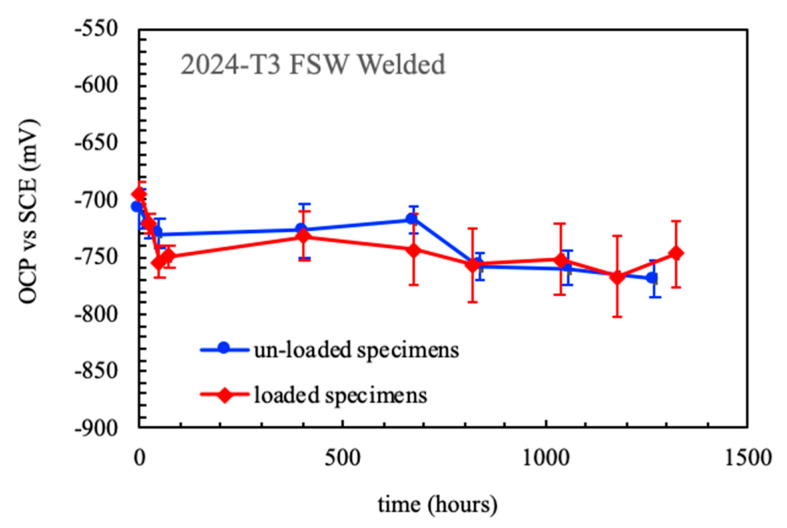
Effect of the time of immersion on the OCP of un-loaded and loaded 4PBB specimens of the FWSed alloy AA 2024-T3 in aerated NaCl 0.6 M at 23 °C (data obtained by [22]).

These results are in agreement with those obtained in previous works [14,22,24]. It was observed that the OCP did not change in four point bent beam (4PBB) specimens in un-loaded or loaded conditions at the 80% of the ultimate tensile strength of the weld (Figure 11). The specimens were loaded before the immersion. On the contrary, differences between the EIS spectra of un-loaded and loaded specimens were observed only at very early exposures. This was mainly attributed to the presence of corrosion products scale formed on the surface of the specimens, that hindered the EIS signal response. On 4PBB specimens, the corrosion process was mainly localized at the nugget, with wide and shallow deep morphology on the un-loaded specimens and stress-enhanced intergranular morphology on loaded specimens [22]. The effect of the applied load on intergranular corrosion initiation and propagation in alloy AA-2424 T3 was first highlighted by Zhang and Frankel [25,26]. However, the entire surface of the specimens is covered with a continuous but porous layer of corrosion products as the exposure time increases. This is well evident in the EIS spectra that are practically overlapped as they are mainly governed by diffusive phenomena occurring at the corrosion products scale [22].

**Figure 12:**
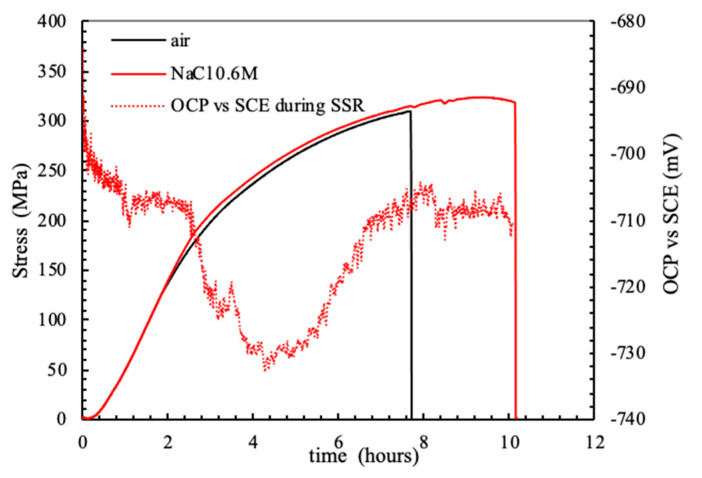
Stress vs. strain and OCP FSWed AA 2024-T3 alloy during the SSR test in aerated NaCl 0.6 M solution at 23 °C.

**Figure 13:**
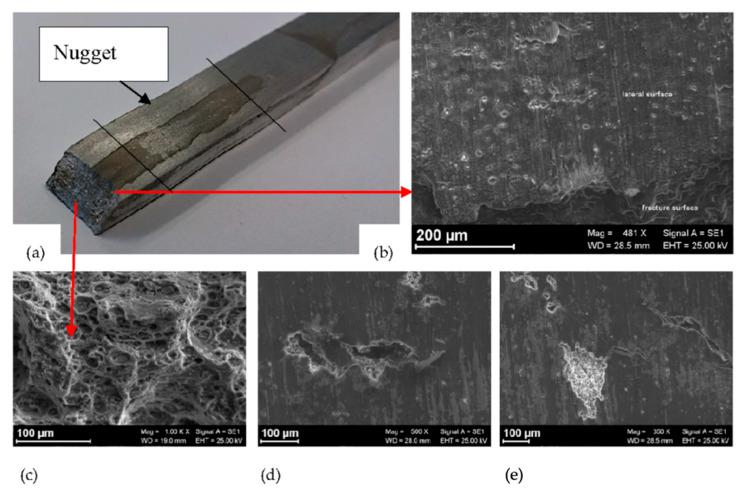
(**a**) Image of a FSWed specimen after the SSR test in NaCl 35 g/L; (**b**) close-up of the lateral surface in the correspondence of the fracture surface; (**c**) close-up of the dimples in the fully ductile fracture surface; (**d**) and (**e**) SCC microcracks in the nugget of the specimen of the FWSed AA 2024-T3.

The OCP of the FSWed AA-2024 T3 alloy under slow strain rate conditions, (Figure 12) modifies in function of the applied monotonic tensile stress. A slight decrease in the values can be noticed as the strain values lay in the elastic field. A sharp decrease was noticed—i.e., 100–250 mV—at the YS. Exceeding the YS, the OCP increases up to the values recorded in the elastic field, until the rupture. The specimen breaking did not occur at the nugget that is characterized by higher tensile strength, but in the heat affected zone. Because of this consideration, it can be derived that the attack observed at the nugget (Figure 13) did not reach sufficient depth to promote the specimen rupture, as the plastic deformation mainly take place in the softened zones, typical of the HAZ/TMAZ. Consequently, the effect of FSW on the alloy microstructure and on the mechanical properties significantly affect also the stress corrosion cracking susceptibility of the alloy. 

**Figure 14:**
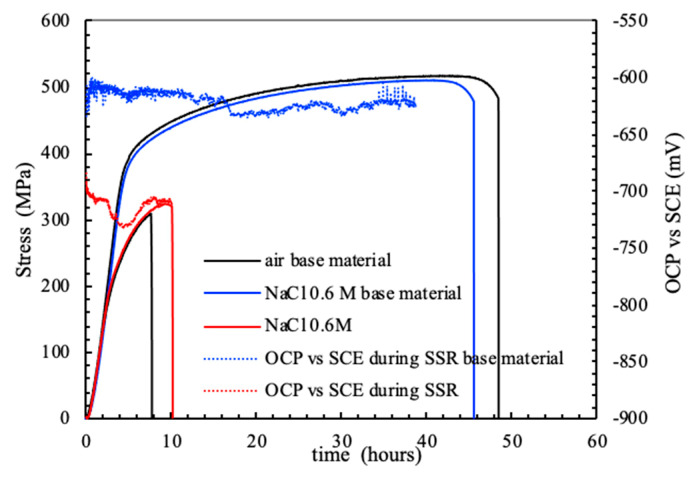
Stress vs. strain and OCP of the base and FSWed AA-2024 T3 alloy during the SSR test in aerated NaCl 0.6 M solution at 23 °C.

SSR tests performed on commercial AA-2024 T3 alloy showed a completely different behavior compared to FSWed. The OCP values are stable at more noble values compared to FSWed, without the occurrence of stress corrosion cracks (Figure 14).

For both the weld and the commercial AA-2024 T3 alloy, the time-to-failure is longer in NaCl solution compared to air. All the tests were twice repeated with similar results. It could be hypothesized that active corrosion in NaCl solution is stimulated at the dislocations, thus generating vacancies able to accelerate the intrinsic mobility and thus the plastic deformation, as already evidenced by other authors that named this phenomenon as anodic attenuation of strain hardening [27,28].

**Figure 15:**
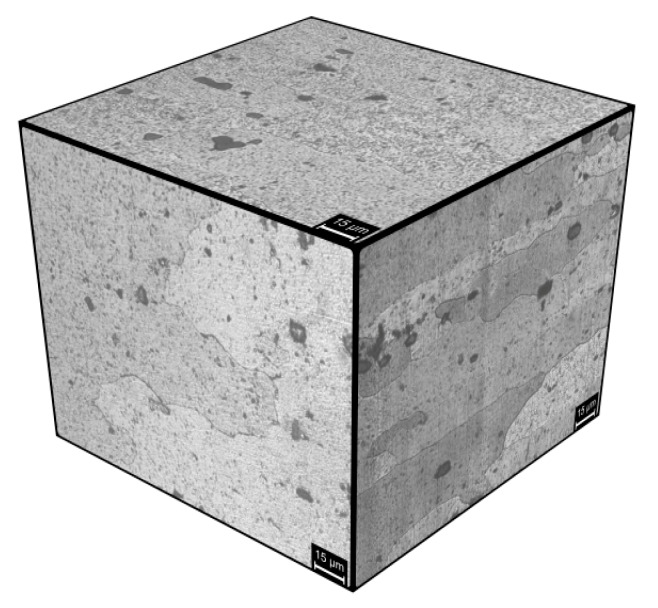
Microstructure of the AA 2024-T3 alloy (Keller attack).

FSW modify the microstructure of the alloy and its electrochemical and stress corrosion cracking behavior, consequently. In the base material, both macro-precipitates and nanoprecipitates are present (Figure 15) [9,11]. The last ones are produced because of solution annealing and natural aging. The macro-precipitates are mainly produced by primary solidification processes and are not solubilized during heat treatment [29]. They are considered to be responsible of the short-term electrochemical behavior of the alloy as it is well-known that the interface between these particles and the matrix is particularly active because of its different practical nobility [30].

The main second phases of AA-2024 T3 alloy are S phases (Al_2_CuMg) and Al-Cu-Mn-Fe [30,31]. Generally, the formers are anodic with respect to the aluminum matrix because of the presence of magnesium, while the latter, consisting of more noble elements such as copper, iron, and manganese, are cathodic. S phase is particularly active and higher amount of this phase is generally present. It quickly reacts with the solution just after dipping, thus leading to the dissolution of magnesium. As a result, the alloy surface becomes richer in copper and the corrosion potential tends to increase. At longer exposures, corrosion initiation occurs in some areas, then propagate in the aluminum matrix. Thick layer of insulating corrosion products then forms on the metal surface, which hinder the corrosion process as a result of the settlement of pseudo-passivation conditions. EIS spectra well evidence the formation of the corrosion products layer as a diffusive behavior at very low frequencies that is compatible with the hypothesized mechanism. The rate-determining step of the corrosion process is then mainly related to the diffusion processes inside the porous non-conductive scale. The phase peaks at low frequencies can be related to the dissolution of the intermetallics at metal/particle interface [32]. 

**Figure 16:**
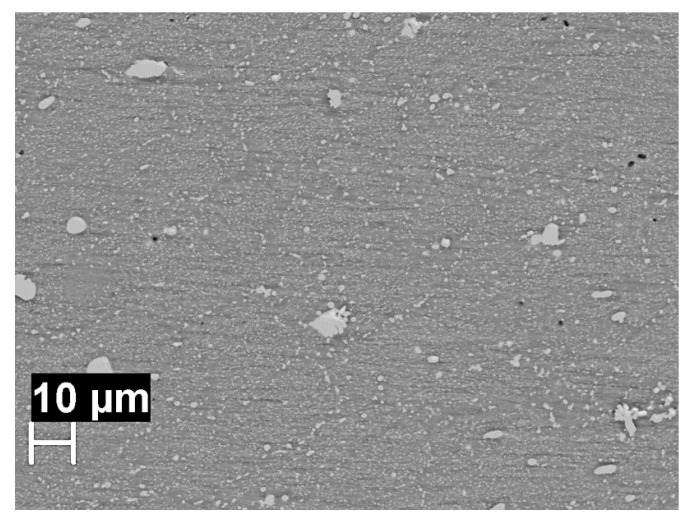
SEM image of the nugget without metallographic attack: the bright zones are the macro and micrometric precipitates of S-phase.

During the welding process, recrystallization induced by the thermomechanical action occurs mainly at the nugget. In these zones, dissolution of nanometric S phase precipitates occurs, which can re-precipitate in form of micrometric particles, clearly detectable at the scanning electron microscope (Figure 16). These precipitates are mainly located at the recrystallized grain boundaries. The presence of copper in the supersaturated solid solution in the aluminum matrix gives rise to initially nobler OCP values compared to the nugget and HAZ. The enriching in copper because of the preferential dissolution of magnesium in S-phase, is responsible for the initial increasing of the OCP, but the rapid activation of corrosion phenomena at precipitate-free zones causes the decrease in the OCP after a few hours of immersion.

The anodic dissolution, catalyzed also by the presence of precipitates along the grain boundaries, is considered to be the main SCC mechanism in high strength 2xxx (Al–Cu–Mg) series alloys [33]. The galvanic interaction between the particles and the surrounding matrix causes the preferential dissolution of the anodic particles or the dissolution of the adjacent matrix surrounding nobler particles. Evolution of hydrogen always occurs in actively growing localized corrosion sites and cracks. The absorption of hydrogen into metals leads to the possibility of crack propagation by hydrogen embrittlement (HE) [33,34,35,36,37,38,39,40,41,42,43,44] 

In this way, FSW modifies the microstructure of AA 2024-T3 alloy, thus making the nugget more susceptible to SCC than the base material. However, the HAZ and TMAZ are characterized by lower tensile strength compared to both the nugget and the base metal. The application of load causes the occurrence of microcracks at the nugget which do not propagate as the fracture is localized in zones with lower tensile strength, i.e., plastic deformation is significantly shifted in the HAZ/TMAZ. Therefore, the strain at the nugget is limited and pseudo-passive film can be restored in such conditions, as demonstrated by the increases in the OCP. 

Similar effect was observed on the same alloy by Ferri et al. on 4PBB loaded in elastic field during potentiodynamic tests [45]. This was attributed to dissolution mechanism assisted by the mechanical stress, according to the chemomechanical theory of Gutman [46]. The weakening of the effect as load levels approach the YS was ascribed to a barrier action of corrosion products film hindering the exit of dislocations from the surface and thus inhibiting the creation of fresh surfaces [45,47]. The stress enhanced intergranular corrosion at nugget is inhibited as the specimens are plastically strained in TMAM/HAZ zones. In this way, the SCC phenomena cannot be observed, and the specimen fails in a ductile way in the TMAZ/HAZ, similarly to the test in air.

In other words, the decreasing of tensile properties in TMAZ/HAZ caused a deformation in these zones, counteracting the hypothetically higher stress enhanced intergranular corrosion susceptibility of the nugget. 

A similar SCC behavior for joint and base metal of FSWed AA2219-T87 through slow strain rate tests (SSR) is reported by Paglia and Buchheit [48]. On the contrary, intergranular fracture in the interface between the nuggets and TMAZ of welded joints tested in 0.6 M NaCl solution is reported by Wang et al. [23]. The last authors reported that the tests in air showed ductile fracture in the same zone. This different behavior could be attributed to the different welding parameters. The same authors reported that the stress corrosion cracking (SCC) susceptibility of the FSW 2024-T4 aluminum alloy joint increased with feed rate due to the increase of the second phases size.

## 4. Conclusions

The paper analyzed stress corrosion cracking phenomena in friction stir welding AA-2024 T3 joints. Constant load (CL) cell and slow strain rate (SSR) tests were carried out in aerated 35 g/L NaCl solution. During the tests, open circuit potential and electrochemical impedance spectroscopy were measured in the different zones of the welding. 

The FSW process significantly modifies the microstructure of the alloy, and therefore, its corrosion and stress corrosion cracking behavior. 

The applied load enhances the intergranular corrosion at the nugget of the AA 2024-T3 alloy owing to the presence of micrometric copper-rich precipitates at the border of the recrystallized grains. In this zones, no stress corrosion cracking was noticed, but well-evident stress-enhanced intergranular corrosion occurred. Moreover, the applied strain is preferentially localized at HAZ/TMAZ zones which are characterized by lower tensile strength compared to the nugget. Higher strain values are then localized at the heat-affected zones, where softening occurs. 

Because of these contrasting effects, it is possible to state that the stress corrosion cracking susceptibility of the FSW-ed alloy is then similar to that of the base metal in the range of processing parameter studied in this experimental work.

## Figures and Tables

**Table 1:** Mechanical properties (yield strength, YS and ultimate tensile strength, UTS) of the base materials and the welded joint.

Alloy	Base Material			FSW Joint	
	YS [MPa]	UTS [MPa]	Max Strain [%]	YS [MPa]	UTS [MPa]
AA 2024–T3	345	459	17	-	315
